# Effect of short-term isometric handgrip training on blood pressure in middle-aged females

**DOI:** 10.5830/CVJA-2010-090

**Published:** 2011-10

**Authors:** Jolene Mortimer, Andrew J Mckune

**Affiliations:** Discipline of Sports Science, School of Physiotherapy, Sports Science and Optometry, Faculty of Health Sciences, University of KwaZulu-Natal, Durban, South Africa; Discipline of Sports Science, School of Physiotherapy, Sports Science and Optometry, Faculty of Health Sciences, University of KwaZulu-Natal, Durban, South Africa

**Keywords:** isometric exercise, blood pressure, females, middle-aged

## Abstract

**Objective:**

To determine the effect of isometric handgrip training on blood pressure (BP) in middle-aged women (47.88 ± 1.8 years).

**Methods:**

Isometric handgrip training was performed over five consecutive days. In each session, the treatment group (*n* = 9) performed four isometric contractions of 45 seconds each at 30% of their maximal grip strength. The control group (*n* = 9) sat for 15 minutes without exercising, for five consecutive days. Resting systolic (SBP) and diastolic blood pressure (DBP) were measured pre- and post-intervention. Data were analysed using a two-factor ANOVA (*p* ≤ 0.05).

**Results:**

Blood pressure readings were reduced in both groups (SBP: *p* = 0.036; DBP: *p* = 0.0079), however there was no interaction effect for SBP or DBP.

**Conclusions:**

The findings suggest that 15 minutes of sitting per day for five consecutive days is just as effective as isometric handgrip training for reducing BP levels. Future research is required to investigate the optimal isometric handgrip training stimulus required to reduce resting BP levels.

## Summary

Hypertension is defined as having a systolic blood pressure (SBP) of 140 mmHg or more, and/or a diastolic blood pressure (DBP) of 90 mmHg or more.^1^ Thirty per cent of the global population is believed to suffer from hypertension.^2^ It is also believed to be the cause of 7.1 million deaths per year, and hypertension is expected to have an increased prevalence of 60% by the year 2025.^3^ In South Africa, research has found that about six million South Africans have hypertension.^4^

Even mild stages of hypertension have been shown to increase the risk of developing more severe hypertension and cardiovascular disease.^5^ Increases in BP of 20/10 mmHg double the risk of cardiovascular disease, while reducing SBP by 3 mmHg decreases the risk of coronary heart disease by 5–9%, and stroke by 8–14%.^6^ The Framingham Heart Study revealed that reducing DBP by 2 mmHg reduced coronary heart disease by 9% and stroke incidents by 15%. A 7.5-mmHg decrease in DBP reduced coronary heart disease by 29% and stroke incidents by 48%.^6^ This clearly indicates the benefit of reducing BP levels.

Traditional treatment of hypertension follows the use of pharmacological agents and lifestyle changes (aerobic exercise, diet, stopping smoking, losing weight, managing stress, and reducing and limiting the amount of sodium and alcohol consumed).^6^ It has been found that the use of pharmacological agents as treatment for hypertension is successful in only about 53% of cases in the USA.^2^ Aerobic exercise (e.g. walking, running, cycling and swimming) has previously been recommended by the American College of Sports Medicine as the primary exercise intervention to prevent and treat hypertension, supplementing this with resistance exercise.^6^

In addition to aerobic and resistance training, static (isometric) exercise has been suggested as an alternative exercise intervention to treat hypertension.^2^ However, the American College of Sports Medicine has not provided any guidelines with regard to isometric training and hypertension.^6^

Previous studies on the hypotensive effects of isometric exercise found that whole-body isometric contractions (using the arms and legs) chronically reduced BP levels.^7^,^8^ Subsequent studies have revealed decreases in BP following participation of hypertensives in isometric handgrip exercise programmes, and return to pre-exercise BP levels following cessation of such programmes.^9^,^10^ Further studies have been performed on medicated hypertensives, on unmedicated hypertensives, and on young normotensive patients.^1^,^2^,^11^

Recently, Millar and associates assessed the effects of isometric handgrip training (using programmed digital hand dynamometers) on medicated hypertensives and determined whether inexpensive spring-loaded handgrips elicited the same response.^2^ These studies found decreases in both SBP and DBP when individuals participated in an isometric handgrip exercise regime. The extent of the decrease differed depending on the intervention variables, including: force of contraction, frequency of exercise (three, four or five days per week), and duration of intervention (five, six or eight weeks).^2^

The greatest average decrease in SBP (156 ± 9.4 to 137 ± 7.8 mmHg, *p* < 0.0005) was recorded by Taylor et al. (2003) who had medicated hypertensives perform four two-minute isometric handgrip contractions with one-minute rest periods in between, three days per week for 10 weeks.^9^ The greatest average reduction in DBP (86.5 ± 2.01 to 71.6 ± 3.50 mmHg, *p* < 0.0001) was observed by Wiley *et al*. (1992) who had the participants (with high-normal DBP) perform four two-minute isometric handgrip contractions at 30% of maximal voluntary contraction, three days a week for eight weeks (24 sessions), with three-minute rest periods in between contractions.^10^

Studies investigating the health benefits of short-term exercise protocols (≤ 10 days of exercise training) are becoming more popular.^12^-^14^ There are, however, no studies that have investigated the effect of a short-term (e.g. a five-day) isometric handgrip training protocol on BP levels.

Elevations in BP occur in both men and women with increasing age, however such increases seem to be greater among post-menopausal women. None of the above studies have investigated the effects of isometric handgrip training on BP levels in middle-aged females (40–60 years old). These studies have also lacked a control group that does not perform any isometric handgrip training.

The purpose of this study was, therefore, to (1) determine the effects of short-term isometric handgrip training on BP in middle-aged females, and (2) compare these results to a control group who would not be performing any isometric contractions. Based on past research showing general BP decreases following isometric handgrip training, it was hypothesised that performing isometric handgrip contractions for 180 seconds a day, for five consecutive days, at 30% of maximal voluntary contraction would reduce the resting BP levels of middle-aged women.

## Methods

Eighteen middle-aged women who were untrained yet physically active were recruited telephonically (Table 1). Physical activity status was determined using the FIT index of Kasari.^15^ This index requires that points are allocated depending on the frequency, intensity and time spent performing physical activity per week, with scores ranging from one (minimum activity) to 100 (training at a high intensity every day of the week). Individuals were included in the study if their FIT index ranged from 8 to 10. This would describe individuals who perform a few days of moderate-intensity physical activity per month, with the duration of physical activity ranging from 20 to 30 minutes per session.

**Table 1:** Participant Characteristics (Mean ± SE) And *p*-Value For Treatment Versus Control

*Variables*	*Treatment (n = 9)*	*Control (n = 9)*	p*-value*
Age	47.88 ± 1.8	49.88 ± 1.4	0.47
Body mass	63.91 ± 3.6	71.80 ± 5.3	0.32
Body mass index	24.92 ± 1.3	27.26 ± 0.8	0.15
Waist–hip ratio	0.76 ± 0.02	0.77 ± 0.02	0.89
Resting HR	67.75 ± 4.5	74.50 ± 3.5	0.25
SBP: pre-	123.0 ± 9.1	130.6 ± 6.4	0.50
DBP: pre-	78.00 ± 5.1	83.88 ± 4.1	0.39
Aerobic capacity	33.93 ± 2.30	34.27 ± 1.30	0.90
FIT index	32.63 ± 8.43	28.88 ± 8.20	0.94
MVC (right): pre-	23.38 ± 1.24	29.25 ± 1.72	0.02*
MVC (left): pre-	23.00 ± 1.38	27.50 ± 1.64	0.05*
MVC (right): post-	25.63 ± 1.10	30.63 ± 2.17	0.06
MVC (left): post-	24.13 ± 1.50	29.38 ± 1.88	0.05*

MVC: maximal voluntary contraction.

Participation was voluntary, and written informed consent was obtained from all participants. The Institution’s Faculty of Health Sciences Ethics Committee approved this study. Participants were excluded from the study if they were receiving pharmacological treatment for hypertension, or tricyclic anti-depressant medication, if they had heart and/or metabolic disease (congestive heart failure, diabetes), or had an exaggerated BP response to exercise (≥ 40 mmHg systolic, and/or ≥ 20 mmHg diastolic following performance of an isometric handgrip maximal contraction).

The study included initial screening of the participants. They were asked to complete a medical history form (including demographic information, information relating to their hypertension, and reproduction, menstrual and family history). Participants were also asked to continue with their normal daily routines for the duration of the study. The pre- and post-testing as well as the intervention were performed in a human performance laboratory. The laboratory temperature (20–22°C) and humidity (50–60%) were kept constant throughout the study.

Pre-testing took place on a Thursday morning between 08:30 and 11:30. Testing involved baseline measures of height, body mass, and waist and hip circumference. Body mass index (BMI) and the waist-to-hip ratio (WHR) were then calculated from these measures. After five minutes of rest, seated resting BP (ausculatory method, using a calibrated aneroid sphygmomanometer), and seated resting heart rate (HR) (using a Suunto T6 heart rate monitor) were recorded. Blood pressure measurement was performed according to the recommendations of the American Heart Association.^16^

Following resting BP and HR measurement, a maximal voluntary contraction (grip strength) test was performed using each hand (right and left). This value was used to determine the appropriate magnitude of contraction (set at 30% maximal voluntary contraction) required during the intervention. A sub-maximal treadmill test was then performed (single-stage treadmill test) to estimate each participant’s aerobic capacity.^17^ Participants were randomly assigned to either the isometric handgrip training group (*n* = 9) or the control group (*n* = 9).

The intervention was performed on five consecutive days of the week (Monday to Friday) (session duration ~15 minutes/session) for both groups. The treatment group had their seated resting HR and BP measured after five minutes of rest. They were then required to perform an isometric handgrip contraction with one hand for 45 seconds at 30% of maximal voluntary contraction. A period of one minute followed this to act as a rest period. An isometric contraction using the other hand was then performed (at 30% maximal voluntary contraction) for 45 seconds. A one-minute rest period followed, and this procedure was repeated, resulting in four isometric contractions held for 45 seconds (two contractions per hand). This made the total duration of exercise 180 seconds (three minutes) per session. Five sessions made the total exercise duration of the treatment group for the entire study 15 minutes.

BP and HR measurements were recorded immediately before the first 45-second contraction, and immediately after the fourth 45-second contraction. This indicated the acute response to the treatment. After sitting for three minutes, HR and BP were measured again.

Upon arrival at the human performance laboratory, the control group was required to sit for five minutes. Their resting BP and HR were then recorded, followed by an additional five minutes of sitting, and subsequent recording of BP and HR. The control group did not perform any isometric contractions.

Post-testing was performed on the Monday morning (08:30 and 11:30) following the last intervention session (Friday). All pre-test measurements were repeated in the post-testing session.

## Statistical analysis

Data were analysed using a two-factor (group *x* time) ANOVA with a Tukey *post-hoc* test to determine specific differences. A *t*-test was used to compare the demographic characteristics of the groups. An alpha level of less than 5% (*p* ≤ 0.05) was considered statistically significant. All data are presented as means ± SE.

## Results

There was no significant baseline difference between the groups in terms of age, body mass, BMI, FIT index, waist-to-hip ratio, resting heart rate, aerobic capacity, SBP and DBP. However, there were significant differences between the treatment and control groups for maximal voluntary contraction pre- and post-intervention (Table 1).

**Fig. 1:**
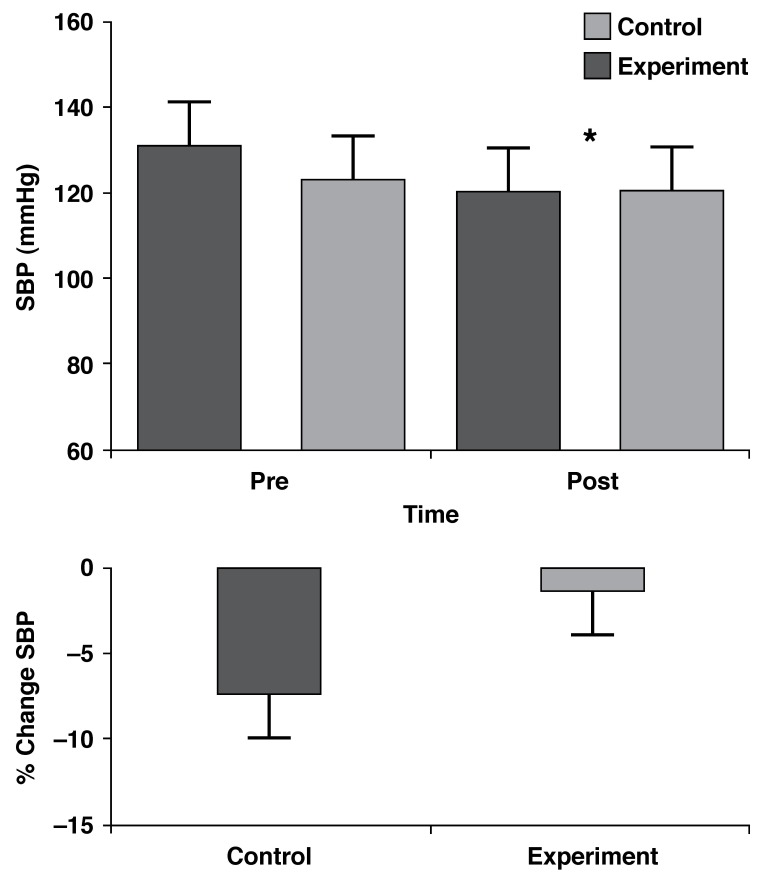
SBP time responses (mean ± SE) and percentage changes of the control (*n* = 9) and treatment (*n* = 9) groups. *Time effect, *p* = 0.036.

For SBP, there was no interaction effect between the treatment and control groups (*p* = 0.17), however there was a significant time effect (*p* = 0.036), with SBP being lower post-intervention. The treatment group showed a reduction from 123 ± 9.1 to 120.6 ± 7.3 mmHg, while the control group showed a reduction from 130.6 ± 6.4 to 120.3 ± 4.8 mmHg (Fig. 1). There was, however, no significant difference in the percentage change in SBP between the two groups (*p* = 0.26) (Fig. 1).

**Fig. 2:**
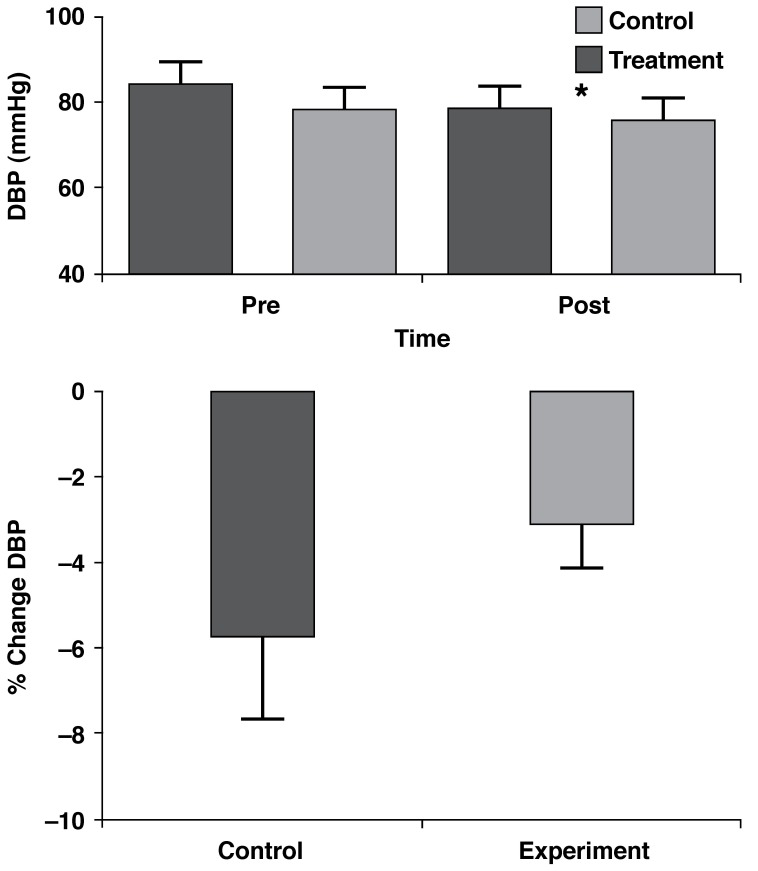
DBP time responses (mean ± SE) and percentage changes of the control (*n* = 9) and treatment (*n* = 9) groups. *Time effect, *p* = 0.0079.

For DBP, there was no interaction effect between the treatment and control groups (*p* = 0.25), however there was a very significant time effect (*p* = 0.0079) with DBP being lower postintervention. The treatment group showed a reduction from 78 ± 5.1 to 75.5 ± 4.9 mmHg, while the control group showed a reduction from 83.88 ± 4.1 to 78.3 ± 4.2 mmHg (Fig. 2). There was, however, no significant difference in the percentage change in DBP between the two groups (*p* = 0.31) (Fig. 2).

## Discussion

The main finding of this study was that the middle-aged women experienced a significant reduction in resting systolic and diastolic blood pressure in response to five consecutive days of laboratory monitoring, regardless of which group they were in (control or treatment). The findings are contrary to previous studies, which reported the hypotensive effects of isometric handgrip training, although in most previous studies, a control group was not used. Since both groups experienced reductions in BP, the hypothesised hypotensive mechanisms of isometric handgrip exercise may not apply to the short-term isometric handgrip-training protocol used in the present study.

A unique finding of the study was that sitting quietly for 15 minutes a day for five consecutive days, whether performing isometric handgrip training or not, had a lowering effect on resting blood pressure. Numerous studies have focused on the effects of relaxation techniques on BP. A study performed by Peters *et al.* (1977) found that relaxation techniques, as well as ‘sitting quietly’ (when performed twice daily for 15 minutes each), elicited BP reductions.^18^ Individuals who were taught the relaxation techniques, however, had greater BP reductions.^18^ In addition, the relaxation-technique group had greater reductions over time (over a 12-week period).^18^ Possible reasons for these reductions may have been related to a decreased activation of the hypothalamic–pituitary–adrenal axis and/or the sympathetic nervous system associated with the relaxation technique.^19^,^20^

McGrady *et al*. (1987) investigated the relationship between cortisol, the relaxation response and high BP. The author found a significant effect of relaxation training on lowering hypothalamic–pituitary–adrenal axis activation, which was associated with a reduction in BP levels.^20^ Albright *et al*. (1991) examined the effect of a stress-management programme on BP levels and found that baseline measures of systolic and diastolic blood pressure decreased significantly after participation in the programme.^19^ In addition, reactivity to a psychological stressor (oral quiz) was significantly lower, as revealed by reduced systolic and diastolic pressure. A reduction in sympathetic nervous system activity was postulated as a possible mechanism for the changes observed.^19^ Further research is required to examine the association between resting BP measurements and alterations in the hypothalamic–pituitary–adrenal axis and/or sympathetic nervous system in middle-aged women.

The participants’ familiarity with the laboratory environment may have accounted for the reductions in BP in the present study, however, research has found that familiarity does not play a major part in BP reductions in long-term follow-up studies of hypertensives.^21^ Short-term follow-up studies (such as this study) have not been investigated in terms of familiarity.

In white-coat hypertension, blood pressure is usually persistently elevated in the presence of a healthcare worker, particularly a physician, however, when measured elsewhere, including while at work, the blood pressure is not elevated.^16^ Although it can occur at any age, this is more common in older men and women.^16^ It is possible that white-coat hypertension may have caused pre-testing BP values to be higher in our study.^22^ The combination of this, followed by increased familiarity with the laboratory environment may have caused the reductions seen in the present study.

An important practical recommendation based on the findings is that future studies aiming to investigate BP should include a minimum of five days of BP monitoring, so as to (1) maximise the effect of familiarity on BP readings once the study commences; (2) minimise the effects of white-coat hypertension on BP readings upon commencement of the study; and (3) allow for any other effects to take their course before the study commences. This would help eliminate bias that may be ‘injected’ into the study through such effects.

## Conclusion

This was a short-term study, which may have accounted for the similar magnitude of reductions in BP in the two groups. The effect of isometric handgrip training may possibly only occur over longer time periods (as supported by previous literature). Future research should therefore investigate the effect of isometric handgrip training over extended time periods using a non-exercise control group. Future studies should also investigate such an effect on hypertensive individuals (stage I hypertension, for example), as any changes in BP with training could be relative to the initial resting BP level.
